# Electroencephalogram-Based Motor Imagery Classification Using Deep Residual Convolutional Networks

**DOI:** 10.3389/fnins.2021.774857

**Published:** 2021-11-17

**Authors:** Jing-Shan Huang, Wan-Shan Liu, Bin Yao, Zhan-Xiang Wang, Si-Fang Chen, Wei-Fang Sun

**Affiliations:** ^1^School of Aerospace Engineering, Xiamen University, Xiamen, China; ^2^Shenzhen Research Institute of Xiamen University, Shenzhen, China; ^3^Institute of Neurosurgery, School of Medicine, Xiamen University, Xiamen, China; ^4^Xiamen Key Laboratory of Brain Center, The First Affiliated Hospital of Xiamen University, Xiamen, China; ^5^Department of Neurosurgery, The First Affiliated Hospital of Xiamen University, Xiamen, China; ^6^College of Mechanical and Electrical Engineering, Wenzhou University, Wenzhou, China

**Keywords:** electroencephalogram (EEG), motor imagery (MI), wavelet packet decomposition (WPD), residual, convolutional neural networks

## Abstract

The classification of electroencephalogram (EEG) signals is of significant importance in brain-computer interface (BCI) systems. Aiming to achieve intelligent classification of motor imagery EEG types with high accuracy, a classification methodology using the wavelet packet decomposition (WPD) and the proposed deep residual convolutional networks (DRes-CNN) is proposed. Firstly, EEG waveforms are segmented into sub-signals. Then the EEG signal features are obtained through the WPD algorithm, and some selected wavelet coefficients are retained and reconstructed into EEG signals in their respective frequency bands. Subsequently, the reconstructed EEG signals were utilized as input of the proposed deep residual convolutional networks to classify EEG signals. Finally, EEG types of motor imagination are classified by the DRes-CNN classifier intelligently. The datasets from BCI Competition were used to test the performance of the proposed deep learning classifier. Classification experiments show that the average recognition accuracy of this method reaches 98.76%. The proposed method can be further applied to the BCI system of motor imagination control.

## Introduction

Electroencephalogram (EEG) is a common biological signal in the medical field. People obtain EEG signals by collecting and recording the potential changes of the superficial skin of the head, and characterize the activity characteristics of the brain. The research of EEG signals is widely used in various aspects. In the field of biomedicine, EEG signal researches help doctors diagnose neurological diseases, such as frostbite, epilepsy, Alzheimer’s disease, childhood developmental disorders, schizophrenia, Parkinson’s disease and other functional diseases. EEG can detect sleep quality, fatigue driving, and drunk driving. It can also study human brain functions such as emotion, cognition, memory, and sports. At present, a mainstream research direction is to use brain waves to control objects. Through certain processing of EEG signals, certain parts of the body can be controlled to make certain actions. At present, certain research results have been achieved. For example, people invented instruments and tools to facilitate the lives of the disabled. Motor imaging signal is a kind of brain electrical signal, which is often used in brain-computer interface (BCI).

Brain-computer interface technology is a system that does not pass through the normal physiological pathways of the human body, but allows the brain to directly transmit information or control commands to computers or related instruments (Gong, 2014). When the brain performs motor imagination, the corresponding brain regional potential will also change accordingly due to the different imagination of the imagination activity. The corresponding changes in the brain are detected, and the computer is used to convert these detected change signals into instructions to control the lower computer (Cilliers and Van Der Kouwe, 1993). The EEG signal is an important part of the BCI system. The acquisition of motor imagery EEG signals is the first step to realize the operation of the BCI system. Then the EEG signal is processed and decoded. Finally, the EEG signal is translated into “machine language” through the control instruction conversion module to drive the external equipment, so that the purpose of human-computer interaction can be realized.

Pattern recognition of various states of the human body based on brainwave detection is a very popular research topic, and it has produced quite constructive results in many fields. K. Polat proposed a classification method of epileptiform EEG using a hybrid system based on decision tree classifier and fast Fourier transform (Kemal and Salih, 2007). M. V. M. Yeo and X. P. Li used support vector machines for pattern recognition and developed a method to automatically detect the driver’s fatigue driving state (Mervyn et al., 2009). The method recognition accuracy reached 99.3%, and it can reliably predict the transition from alertness to drowsiness. T. Nguyen proposed a threshold method to identify blinking state, and achieved good results in the detection results (Nguyen et al., 2013). Wang proposed an EEG eye state identification method using incremental attribute learning with time-series classification, and the method finally achieved an accuracy rate higher than average (Wang et al., 2014). S. K. Satapathy used neural network and support vector machine to perform brain wave-based pattern recognition for epilepsy and has achieved good recognition results (Satapathy et al., 2017). G. Anumanchipalli uses the RNN deep learning model to directly read the thoughts in the brains of paralyzed patients using a BCI. The spoken sentences can reach 150 words per minute, which is close to the normal level of people (Anumanchipalli et al., 2019).

Since the end of the 1960s, humans have studied BCI technology for more than 50 years. Since the 21st century, the research of BCI has become more and more prosperous. Four international BCI competitions were successfully held. Researchers have systematically analyzed the processing methods of EEG signals and produced some mature applications. The BCI laboratory team at the Cognitive Institute of Graz University of Technology in Austria first implemented a BCI based on online EEG classification. The team developed a variety of BCI systems using motor imaging brain electrical signals, including imagining different limb movements to control the movement of the wheelchair (Pfurtscheller et al., 1993, 2006), and using brain waves to control the movement of the mouse to find coins (Pfurtscheller et al., 2000). The BCI Research Institute in Berlin has developed a typing system (Benjamin et al., 2003). The subjects selected different characters for typing by imagining the movements of the left hand, right hand, and foot. The Washington Research Center in the United States uses different EEG rhythm signals generated by motor imagination to realize the free movement of the virtual cursor in three-dimensional space (McFarland et al., 2010). The BSI-TOYOTA Collaboration Center in Japan has successfully developed a real-time control wheelchair using brain waves. By imagining the front, left, and right to control the direction of the wheelchair, a 125 ms response control system for the electric wheelchair can be realized (Bai, 2010). Gao Shangkai of Tsinghua University used the characteristics of motor imaginary EEG signals to develop a system that uses EEG signals to control robot dogs playing football (Wang et al., 2007). Xu Baoguo of Southeast University controlled the robot arm to make corresponding actions based on imagining the movement of the hand (Xu et al., 2011). The average accuracy of motor imaging EEG for manipulator control is 88%. Li Yuanqing of South China University of Technology designed a hybrid BCI system that combines motor-imaging EEG signals and P300 signals, which control the horizontal and vertical movement of the cursor, respectively (Li et al., 2010).

Although many laboratory results have been achieved in the research of BCI technology, there are still few products that can be applied in real life. The BCI technology is still in the stage of theoretical research and laboratory development, and the application system needs to be further improved. There are still many key technologies that need to be improved. Firstly, the existing BCI systems have poor adaptability. Different individuals have different physiological functions, so people’s EEG physiological responses to the same task will also be different. When the same individual performs the same motor imagination activity in different mental states, the EEG response may also be different. The adaptability of the future BCI system should not only meet the differences between different individuals, but also meet the changes of different states of the same individual. Secondly, the recognition speed and accuracy of the BCI system need to be improved. Classification accuracy and recognition speed are the most commonly performance evaluation indicators used in BCI system. The existing feature extraction methods used for classification of motion imaging tasks are relatively complicated. The large amount of data leads to long calculation time and slow system processing speed. However, reducing the amount of data or simplifying the signal feature extraction method will cause the classification accuracy to decrease. The key challenge of current research is to speed up the processing speed of the system while ensuring the accuracy of classification. In addition, it is relatively difficult to integrate technologies in different fields with BCI system. Promoting the technical integration of BCI application systems and being accepted by users is also an important practical problem faced by BCI systems.

In this article, we propose an accurate EEG signal classification method using Deep Residual Convolutional Neural Network (DRes-CNN). The EEG signals in BCI Competition 2005 data set IVa and BCI Competition 2003 data set III are selected as the original data. The wavelet packet decomposition (WPD) was used for preprocessing to obtain the characteristics of EEG signals. Subsequently, the reconstructed EEG signals of different frequency bands were used as the input of DRes-CNN to finally identify and classify the EEG types. The classification results show that the average accuracy of the proposed DRes-CNN model can reach 98.76%. The rest of this article is organized as follows. In section “Method,” we explained the methods used for EEG classification, including database and segmentation, data preprocessing based on WPD, and the proposed deep residual neural network. In section “Results,” the numerical evaluation and experimental results of the EEG classification are shown. Finally, we give the discussion and conclusion in section “Discussion.”

## Method

### Methodology Overview

The proposed EEG classification method is based on the WPD and the proposed deep residual convolutional networks. The original EEG signals were shared by the BCI Competition database (Blankertz et al., 2006). Firstly, EEG waveforms are segmented into sub-signals. Then the EEG signal features are obtained through the WPD algorithm, and some selected wavelet coefficients are retained and reconstructed into EEG signals in their respective frequency bands. Subsequently, the reconstructed EEG signals were utilized as input of the proposed deep residual convolutional networks to classify EEG signals. Finally, EEG types of motor imagination are classified by the DRes-CNN classifier intelligently.

### Database and Segmentation

The international organizations have held several BCI competitions since 2001. The International BCI Competition provides a reliable data source and a unified test standard for researchers in the field of motor imaging EEG signal analysis. The experimental data in this article comes from the databases in BCI Competition 2005 (dataset IVa) and BCI Competition 2003 (dataset III).

These two databases contain data sets recorded by five subjects (aa, al, av, aw, and ay). All five subjects performed the BCI experiment, which included three exercises of motor imagination for the left hand, right hand, and right foot. In this experiment, only the right hand (R) and right foot (F) two types of motor imagination are used for data analysis, and they are named Class-1 and Class-2. Each EEG signal has 118 channels. These motor imaging tasks are classified by using the EEG signals recorded on the C3, Cz, and C4 channels. At the beginning of the experiment, a prompt appeared in the center of the screen to inform the subject of the motion imaging task to be performed. Each test takes 7 s. During the first 2 s, the subject remained sitting still. At *t* = 2 s, an auditory stimulus will appear, prompting the start of the experiment. At *t* = 3 s, an arrow will appear on the screen to indicate which imaginary exercise the subject is performing. At the same time, the subject began to perform an imaginary movement in the same direction as the arrow prompts. The subject’s imagination time is 3.5 s. After the motion imaging, the subjects had a short rest period, which ranged from 1.75 to 2.25 s. At *t* = 7 s, the arrow disappears and the subject ends the imaginary action. The sampling frequency of EEG is 250 Hz. The EEG waveform is divided into time samples of 3.5 s. There are 140 experimental samples for each type of EEG signal.

### Data Preprocessing *via* Wavelet Packet Decomposition

Electroencephalogram signals have time-varying and non-stationary characteristics. Time domain analysis mainly considers the geometric characteristics of signal variance and mean value, and frequency domain analysis mainly considers the characteristics of signal coherence and frequency band power. EEG signals are constantly changing with time. Neither time domain analysis nor frequency domain analysis alone can accurately reflect its characteristics. Time-domain joint analysis is more suitable for reflecting the transient characteristics of non-stationary signals.

Wavelet packet decomposition (Walczak and Massart, 1997; Manthalkar et al., 2003) is an improved method based on wavelet decomposition. This method makes up for the low resolution of the high-frequency part of WPD. It can analyze the signal more accurately. For different signals, WPD can automatically select the appropriate frequency band to match the frequency spectrum of the signal, thereby improving the time-frequency resolution. WPD has a good performance in signal local analysis. It can effectively remove the redundant information and retain the feature information that is beneficial to classification to best express the EEG signal feature information.

In multi-resolution analysis, WPD is regarded as a process of stepwise orthogonal decomposition of a function space. Multi-resolution analysis decomposes the L^2^(R) of the Hilbert space into the orthogonal sum of all wavelet subspaces **W_l_** according to different scale factors **l**. A new subspace ${\text{U}}_{l}^{m}$ is defined to represent the wavelet subspace **W_l_** and the scale space **V_l_**.


(1) $$\left\{ \begin{array}{c} {\text{U}}_{l}^{0}={\text{V}}_{l},\text{l}\in \text{Z}\\ {\text{U}}_{l}^{1}={\text{W}}_{l},\text{l}\in \text{Z} \end{array}\right.$$

The orthogonal decomposition of Hilbert space **V_l_**⊕**W_l_** can be expressed as:


(2) $${\text{U}}_{l}+1^{0}={\text{U}}_{l}^{0}⊕{\text{U}}_{l},\text{l}\in Z.$$

Define the subspace ${\text{U}}_{l}^{m}$ as a closure space of the function **u_m_**(**t**), so that **u_m_**(**t**) satisfies:


(3) $$\left\{ \begin{array}{c} {\text{u}}_{2\,m}\,\left(t\right)=\sqrt{2}\,\sum_{k\in Z}\text{h}\,\left(k\right)\,{\text{u}}_{2\,m}\,\left(2\,\text{t}-\text{k}\right)\\ {\text{u}}_{2\,m+1}\,\left(t\right)=\sqrt{2}\,\sum_{k\in Z}\text{g}\,\left(k\right)\,{\text{u}}_{2\,m}\,\left(2\,\text{t}-\text{k}\right) \end{array}\right.,$$

where **g**(**k**) = (−−**^1^**)**kh**(**1**−**k**), **g**(**k**), and **h**(**k**) are the coefficients of the high-pass filter and the low-pass filter, which are orthogonal to each other.

When *m* = 0, from Equation 3, we can get:


(4) $$\left\{ \begin{array}{c} {\text{u}}_{0}\,\left(t\right)=\sum_{k\in Z}\text{h}\,\left(k\right)\,{\text{u}}_{0}\,\left(2\,\text{t}-\text{k}\right)\\ {\text{u}}_{1}\,\left(t\right)=\sum_{k\in Z}\text{g}\,\left(k\right)\,{\text{u}}_{0}\,\left(2\,\text{t}-\text{k}\right) \end{array}\right..$$

In the process of multi-resolution analysis, the wavelet basis function *φ*(*t*) and scale function *ψ*(*t*) satisfy:


(5) $$\left\{ \begin{array}{c} \varphi \,\left(t\right)=\sum_{k\in Z}\text{g}\,\left(k\right)\,\psi \,\left(2\,\text{t}-\text{k}\right)\\ \psi \,\left(t\right)=\sum_{k\in Z}\text{h}\,\left(k\right)\,\psi \,\left(2\,\text{t}-\text{k}\right) \end{array}\right..$$

From the Equations 4, and 5, we can know that *φ*(*t*) = *u*_1_(*t*) and *φ*(*t*) = *u*_0_(*t*). Therefore {**u_m_**(**t**)}_***m***∈***Z***_ is an orthogonal wavelet packet. The calculation formula of the WPD coefficient is shown in the Equation 6:


(6) $$\{\begin{array}{c} d_{i}^{l+1,2\,m}=\sum_{k\in Z}\text{h}\,\left(\text{k}-2\,\text{i}\right)\,{\text{d}}_{k}^{l,m}\\ {\text{d}}_{i}^{l+1,2\,m+1}=\sum_{k\in Z}\text{g}\,\left(\text{k}-2\,\text{i}\right)\,{\text{d}}_{k}^{l,m} \end{array}.$$

**FIGURE 1:**
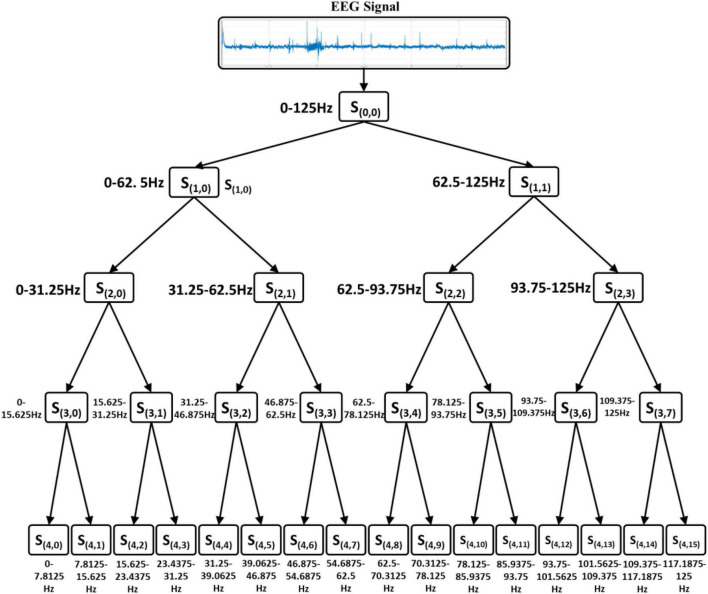
Four-layer wavelet packet decomposition of EEG signal.

The WPD has good time-frequency resolution in both high-frequency and low-frequency parts. This method saves all the energy of the signal, so it is very suitable for the analysis and processing of EEG signals. Since the information of the EEG signal reflected by the wavelet packet coefficients on each decomposition scale is different, it can be considered to extract features from part of the wavelet packet coefficients. In the process of WPD, the decomposition scale and basis function have a great influence on the decomposition effect. The higher the scale of WPD, the better its local characteristics. But the dimensionality of the feature is also larger, which will prolong the training time of the model. Studies have confirmed that the ERD/ERS phenomenon that characterizes motor imagination actions is mainly reflected in the 8–30 Hz of the EEG signal (Pfurtscheller et al., 1997). Therefore, the decomposition scale should ensure that the frequency band corresponding to the wavelet packet coefficient is within the frequency range. Therefore, the decomposition level of WPD is determined to be four. The four-layer WPD of the EEG signal is shown in Figure 1. The signal is divided into 16 frequency bands, and the frequency range corresponding to each node in each layer is shown in Figure 1.

**FIGURE 2:**
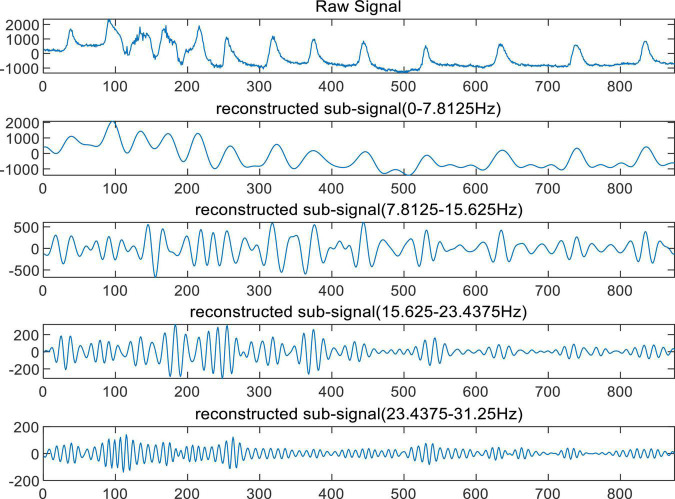
Examples of short EEG recordings and their reconstructed sub recordings.

According to the characteristics of the original EEG signal and the wavelet basis functions, the selected wavelet basis is Db4 (Guan et al., 2015). The original EEG is decomposed by wavelet packet to obtain wavelet coefficients on various scales. The wavelet coefficients of the 0th, 1st, 2nd, and 3rd nodes in the fourth-level decomposition are retained and reconstructed into EEG signals in their respective frequency bands. The reconstructed EEG signals filter out high-frequency noise in the original signal and signals in other frequency bands that are not related to motor imagination. The reconstructed EEG signals of ${\text{S}}_{4}^{0}$, ${\text{S}}_{4}^{1}$, ${\text{S}}_{4}^{2}$, and ${\text{S}}_{4}^{3}$ are used as the input of the EEG classifier. The examples of short EEG recordings and their reconstructed sub recordings are shown in Figure 2.

### The Proposed Deep Residual Convolutional Networks

Convolutional neural network (CNN) is a deep feedforward neural network (Liu, 2018) inspired by the concept of “receptive field.” With the increase of the number of layers and neurons in the deep neural network, the non-linear fitting ability will increase. CNN is widely used in engineering fault diagnosis (Cao et al., 2019), medical signal recognition (Chandra et al., 2019; Huang et al., 2019), image recognition (Qu et al., 2016) and other fields. However, simply stacking the number of network layers will cause the problem of vanishing gradients. The network can be converged by normalizing initialization and introducing a median normalization layer. But for a deeper network, the accuracy of the model will decrease as the depth increases when the network model accuracy reaches saturation. This is the degradation problem of neural networks (He et al., 2016; Yu et al., 2016). The neural network learns an implicit abstract mapping relationship by adjusting its parameters. However, this implicit mapping relationship is difficult to optimize in a deeper network. The purpose of the deep residual convolutional neural network (DRes-CNN) method is to solve the degradation problem of traditional neural networks. The learning process of the DRes-CNN is using multiple consecutively stacked non-linear computing layers to fit the residual F(x) between the input data and the mapped output data. The residual F(x) is calculated as follows:


(7) F⁢(x)=H⁢(x)-x,

where H(x) is the optimal solution, and x is the input congruent mapping.

The closer the residual F(x) is to 0, the closer the features extracted by this network are to the original input. The DRes-CNN composed of the residual block local units can solve the difficulty in convergence and adjustment. It overcomes the degradation problem of CNN as the number of network layers increases.

**FIGURE 3:**
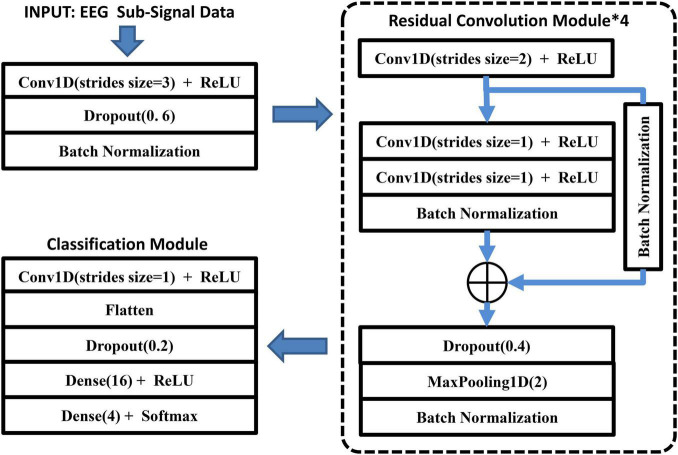
The architecture of the proposed DRes-CNN.

In this section, we propose the deep residual convolutional neural networks (DRes-CNN). As shown in Figure 3, the DRes-CNN is mainly composed of four residual convolution modules and a classification module. In the proposed deep residual convolutional networks, a convolutional layer with a stride of 3, a random dropout layer and a batch-normalization layer are applied firstly to compress the input EEG data and enhance the generalization of the DRes-CNN model. The down-sampling module can effectively simplifies the calculation of deep network models, reduces data redundancy, and promotes model learning (Huang et al., 2020). In the four residual convolution module, convolutional layers in series are followed by residual short circuit. Then a random dropout layer and the max-pooling layer are added after the convolutional layers. Finally, in the classification module, a flatten layer follows the convolution layer and a random dropout layer is applied after the flatten layer to prevent overfitting. In the proposed DRes-CNN model, the learning rate is set as 0.001 and the batch size parameter is set as 250.

## Results

### Evaluation Metrics

The performance of the classification model is mainly measured by the accuracy. The accuracy was calculated through Equation 8.


(8) $$\text{Accuracy}\left(\%\right)=\frac{\text{TP}+\text{TN}}{\text{TP}+\text{TN}+\text{FP}+\text{FN}}\times 100,$$

where TP stands for true positive, meaning the correct classification as Class-1 of EEG; TN stands for true negative, meaning correct classification as Class-2 of EEG; FP stands for false positive, meaning incorrect classification as Class-1 of EEG; FN represents false negative, meaning incorrect classification as Class-2 of EEG (Yin et al., 2016).

### The Experimental Classification Results

In order to verify the effectiveness of the proposed EEG classification model, we classify the EEG signals of the right hand (Class-1) and right foot (Class-2). One hundred forty groups of eight feature inputs can be obtained for each type of EEG signal after data preprocessing based on WPD. All EEG training sample data is randomly scrambled, and the last 200 samples are selected as the test set. The classification of EEG signals is based on the classification algorithm described in section “Method.”

The original EEG waveform is divided into sub-signals. Then, the characteristics of the EEG signal are obtained through the WPD algorithm. The specific wavelet coefficients are retained and reconstructed into the EEG signals of respective frequency bands. Subsequently, the reconstructed EEG signal is used as the input of the proposed deep residual convolutional network to complete the classification of EEG signals. The experiment was run on a PC with 32 GB of memory and 16 GB of GPU memory.

**FIGURE 4:**
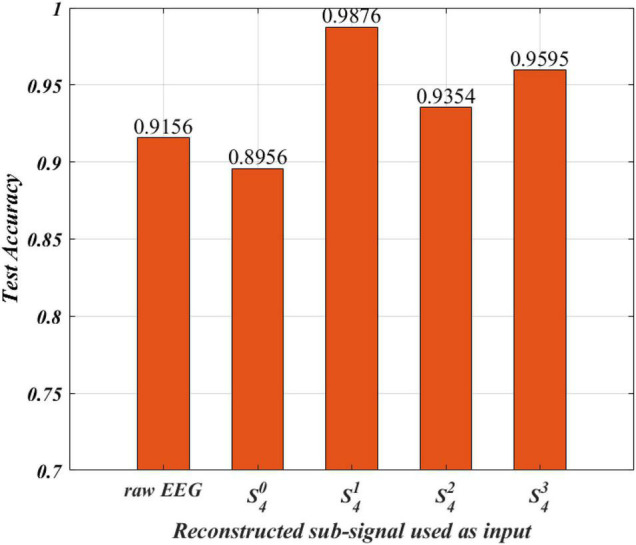
Comparison of average test accuracy by different reconstructed sub-signal.

Electroencephalogram signals of different frequency bands contain different characteristics and information. The wavelet packet can decompose the information of each frequency band. It makes the characteristics of the EEG signal easier to identify in each frequency band. In this section, the performance of training the DRes-CNN with reconstructed sub-signals as model input is studied experimentally, and the experiment results are shown in Figure 4.

From Figure 4, we can find that the accuracy of the classification test using the original EEG signal as input reaches 91.56%. The reconstructed EEG data set of $S_{4}^{0}$ (0, 7.8125 Hz) reached an average test accuracy of 89.56%. The reconstructed EEG data set of $S_{4}^{1}$ (7.8125, 15.625 Hz) reached the best average test accuracy of 98.76%. The reconstructed EEG data of $S_{4}^{2}$ (15.625, 23.4375 Hz) set reached an average test accuracy of 93.54%. The reconstructed EEG data set of $S_{4}^{3}$ (23.4375, 31.25 Hz) reached an average test accuracy of 95.95%. From the experimental comparison demonstrated above, we can conclude that the proposed DRes-CNN model shows the best classification performance when the reconstructed EEG dataset of $S_{4}^{1}$ (7.8125, 15.625 Hz) is used as model input.

## Discussion

In this article, we proposed an EEG classification method using WPD and the proposed deep residual convolutional network. The goal of this method is to achieve high-precision intelligent classification of motor-imaging EEG signals. The original EEG signal is shared by the BCI Competition database. Firstly, the EEG waveform is divided into shorter sub-signals. Then, the characteristics of the EEG signal are obtained through the WPD algorithm. Some selected wavelet coefficients are retained and reconstructed into EEG signals of their respective frequency bands. Subsequently, the reconstructed EEG signal is used as the input of the proposed deep residual convolutional network. Finally, the motor imagery EEG signals are intelligently classified by the DRes-CNN classifier. We compared the classification performance of reconstructed signals in different frequency bands as input to the model. Through comparative experiments, we found that the proposed DRes-CNN model shows the best classification accuracy of 98.76% when the reconstructed EEG data set in the frequency band of (7.8125, 15.625 Hz) is used as the model input. The proposed method can be further applied to the BCI system of motor imagination control.

## Data Availability Statement

The original contributions presented in the study are included in the article/supplementary material, further inquiries can be directed to the corresponding authors.

## Author Contributions

J-SH, W-SL, and BY conceived and designed the classification method. Z-XW and S-FC performed the experiment. J-SH preprocessed and analyzed the data and wrote the manuscript. BY, S-FC, and W-FS reviewed and edited the manuscript. J-SH and W-FS responded to the comments of the reviewers. All authors read and approved the manuscript.

## Conflict of Interest

The authors declare that the research was conducted in the absence of any commercial or financial relationships that could be construed as a potential conflict of interest.

## Publisher’s Note

All claims expressed in this article are solely those of the authors and do not necessarily represent those of their affiliated organizations, or those of the publisher, the editors and the reviewers. Any product that may be evaluated in this article, or claim that may be made by its manufacturer, is not guaranteed or endorsed by the publisher.

## References

[B1] Anumanchipalli G. Chartier J. Chang E. (2019). Speech synthesis from neural decoding of spoken sentences. *Nature* 568 493–498. 10.1038/s41586-019-1119-1 31019317 PMC9714519

[B2] Bai X. (2010). Real-time control of electric wheelchairs by brain waves. *Robot Technol. Appl.* 2 10–12.

[B3] Benjamin B. Guido D. Matthias K. Schroder M. Williamson J. Murray-Smit R. (2003). The Berlin brain-computer interface presents the novel mental typewriter Hex-O-Spell. *Comput. Intell. Neurosci.* 14 332–336.

[B4] Blankertz B. Muller K. R. Krusienski D. J. Schalk G. Wolpaw J. R. Schlögl A. (2006). The BCI competition III: validating alternative approaches to actual BCI problems. *IEEE Trans. Neural Syst. Rehabil. Eng.* 14 153–159. 10.1109/TNSRE.2006.875642 16792282

[B5] Cao X. C. Chen B. Q. Yao B. He W.-P. (2019). Combining translation-invariant wavelet frames and convolutional neural network for intelligent tool wear state identification. *Comput. Ind.* 106 71–84. 10.1016/j.compind.2018.12.018

[B6] Chandra B. S. Sastry C. S. Jana S. (2019). Robust heartbeat detection from multimodal data via CNN-Based generalizable information fusion. *IEEE Trans. Biomed. Eng.* 66 710–717. 10.1109/TBME.2018.2854899 30004868

[B7] Cilliers P. J. Van Der Kouwe A. J. W. (1993). “A VEP-based computer interface for C2-ouadriplegics,” in *Proceedings of the 15th Annual International Conference of the IEEE*, (San Diego, CA: IEEE), 1263–1263. 10.1109/IEMBS.1993.979126

[B8] Gong P. (2014). *Research on Feature Extraction of Motor Imagination EEG Signal.* Chongqing: Chongqing University.

[B9] Guan W. Qingzhi Y. Yang G. (2015). Detection and location of transient power quality disturbances in distribution network based on db4 wavelet. *Power Syst. Prot. Control* 43 102–106.

[B10] He K. Zhang X. Ren S. Sun J. (2016). “Deep residual learning for image recognition,” in *Proceedings of the IEEE Conference on Computer Vision and Pattern Recognition*, Las Vegas, NV, 770–778. 10.1109/CVPR.2016.90

[B11] Huang J. Chen B. Yao B. He W. (2019). ECG arrhythmia classification using STFT-based spectrogram and convolutional neural network. *IEEE Access* 7 92871–92880. 10.1109/ACCESS.2019.2928017

[B12] Huang J. Chen B. Zeng N. Cao X.-C. Li Y. (2020). Accurate classification of ECG arrhythmia using MOWPT enhanced fast compression deep learning networks. *J. Ambient Intell. Humaniz. Comput.* 5 1–18. 10.1007/s12652-020-02110-y

[B13] Kemal P. Salih G. (2007). Classification of epileptiform eeg using a hybrid system based on decision tree classifier and fast fourier transform. *Appl. Math. Comput.* 187 1017–1026. 10.1016/j.amc.2006.09.022

[B14] Li Y. Long J. Yu T. Yu Z. Wang C. Zhang H. (2010). An EEG-based BCI system for 2-D cursor control by combining Mu/Beta rhythm and P300 potential. *IEEE Trans. Biomed. Eng.* 57 495–2505. 10.1109/TBME.2010.2055564 20615806

[B15] Liu C. (2018). *Research and Design of Handwritten Digit Recognition Based on Convolutional Neural Network.* Chengdu: Chengdu University of Technology.

[B16] Manthalkar R. Biswas P. K. Chatterji B. N. (2003). Rotation and scale invariant texture features using discrete wavelet packet transform. *Patt. Recognit. Lett.* 24 2455–2462. 10.1016/S0167-8655(03)00090-4

[B17] McFarland D. J. Sarnacki W. A. Wolpaw J. R. (2010). Electroencephalographic (EEG) control ofthree-dimensional movement. *J. Neural Eng.* 7:036007. 10.1088/1741-2560/7/3/036007 20460690 PMC2907523

[B18] Mervyn V. Li X. Shen K. Wilder-Smith E. P. (2009). Can SVM be used for automatic EEG detection of drowsiness during car driving. *Saf. Sci.* 47 115–124. 10.1016/j.ssci.2008.01.007

[B19] Nguyen T. Nguyen T. Truong K. Toi V. (2013). *A Mean Threshold Algorithm for Human Eye Blinking Detection Using EEG.* Berlin: Springer, 275–279. 10.1007/978-3-642-32183-2_69

[B20] Pfurtscheller G. Flotzinger D. Kalcher J. (1993). Brain-computer interface-a new communication device for handicapped persons. *J. Microcomput. Appl.* 16 293–299. 10.1006/jmca.1993.1030

[B21] Pfurtscheller G. Muller-Putz G. R. Schlogl A. (2006). 15 years of BCI research at graz university of technology : current projects. *IEEE Trans. Neural Syst. Rehabil. Eng.* 14 205–210. 10.1109/TNSRE.2006.875528 16792295

[B22] Pfurtscheller G. Neuper C. Flotzinger D. Pregenzer M. (1997). EEG-based discrimination between imagination of right and left hand movement. *Electroencephalogr. Clin. Neurophysiol.* 103 642–651. 10.1016/S0013-4694(97)00080-1 9546492

[B23] Pfurtscheller G. Neuper C. Guger C. Harkam W. Ramoser H. Schlögl A. (2000). Current trends in Graz brain–computer interface (BCI) research. *IEEE Trans. Rehabil. Eng.* 8 216–219. 10.1109/86.847821 10896192

[B24] Qu J. Xian S. Xin G. (2016). Remote sensing image target recognition based on CNN. *Foreign Electronic Meas. Technol.* 8 45–50.

[B25] Satapathy S. Jagadev A. Dehuri S. (2017). Weighted majority voting based ensemble of classifiers using different machine learning techniques for classification of eeg signal to detect epileptic seizure. *Informatica* 41 99–110.

[B26] Walczak B. Massart D. L. (1997). Noise suppression and signal compression using the wavelet packet transform. *Chemometr. Intell. Lab. Syst.* 36 81–94. 10.1016/S0169-7439(96)00077-9

[B27] Wang T. Guan S. Man K. Ting T. (2014). EEG eye state identification using incremental attribute learning with time-series classification. *Math. Probl. Eng.* 2014:365101. 10.1155/2014/365101

[B28] Wang Y. Hong B. Gao X. Gao S. (2007). Implementation of a Brain-computer interface based on three states of motor imagery. *Annu. Int. Conf. IEEE Eng. Med. Biol. Soc.* 2007 5059–5062. 10.1109/IEMBS.2007.4353477 18003143

[B29] Xu B. Aiguo S. Renhuan Y. (2011). Online brain-computer interface experiment based on motor imagination EEG. *J. Huazhong Univ. Sci. Technol.* 39 60–64.

[B30] Yin W. Yang X. Zhang L. Oki E. (2016). ECG monitoring system integrated with IR-UWB radar based on CNN. *IEEE Access* 4 6344–6351. 10.1109/ACCESS.2016.2608777

[B31] Yu L. Chen H. Dou Q. Qin J. Heng P.-A. (2016). Automated melanoma recognition in dermoscopy images via very deep residual networks. *IEEE Trans. Med. Imaging* 36 994–1004. 10.1109/TMI.2016.2642839 28026754

